# Thoracic radiation-induced pleural effusion and risk factors in patients with lung cancer

**DOI:** 10.18632/oncotarget.18824

**Published:** 2017-06-29

**Authors:** Jing Zhao, Regina M. Day, Jian-Yue Jin, Leslie Quint, Hadyn Williams, Catherine Ferguson, Li Yan, Maurice King, Ahmad Albsheer, Martha Matuszak, Feng-Ming (Spring) Kong

**Affiliations:** ^1^ Department of Oncology, Tongji Hospital, Tongji Medial College, Huazhong University of Science and Technology, Wuhan, Hubei, China; ^2^ Department of Radiation Oncology, Medical College of Georgia, Augusta University, Augusta, GA, USA; ^3^ Department of Pharmacology, Uniformed Services University of the Health Sciences, Bethesda, MD, USA; ^4^ Department of Radiation Oncology, Radiation Physics, Indiana University School of Medicine, Indianapolis, IN, USA; ^5^ Department of Radiology, University of Michigan, Ann Arbor, MI, USA; ^6^ Department of Radiology, Medical College of Georgia, Augusta University, Augusta, GA, USA; ^7^ Department of Radiation Oncology, University of Michigan, Ann Arbor, MI, USA; ^8^ Department of Radiation Oncology, IU Simon Cancer Center, Indiana University School of Medicine, Indianapolis, IN, USA

**Keywords:** lung cancer, thoracic radiotherapy, radiation induced pleural effusion, risk factors, overall survival

## Abstract

The risk factors and potential practice implications of radiation-induced pleural effusion (RIPE) are undefined. This study examined lung cancer patients treated with thoracic radiation therapy (TRT) having follow-up computed tomography (CT) or 18F-fluorodeoxyglucose (FDG) positron emission tomography (PET)/CT. Increased volumes of pleural effusion after TRT without evidence of tumor progression was considered RIPE. Parameters of lung dose-volume histogram including percent volumes irradiated with 5-55 Gy (V5-V55) and mean lung dose (MLD) were analyzed by receiver operating characteristic analysis. Clinical and treatment-related risk factors were detected by univariate and multivariate analyses. 175 out of 806 patients receiving TRT with post-treatment imaging were included. 51 patients (24.9%) developed RIPE; 40 had symptomatic RIPE including chest pain (47.1%), cough (23.5%) and dyspnea (35.3%). Female (OR = 0.380, 95% CI: 0.156–0.926, *p* = 0.033) and Caucasian race (OR = 3.519, 95% CI: 1.327–9.336, *p* = 0.011) were significantly associated with lower risk of RIPE. Stage and concurrent chemotherapy had borderline significance (OR = 1.665, *p* = 0.069 and OR = 2.580, *p* = 0.080, respectively) for RIPE. Patients with RIPE had significantly higher whole lung V5-V40, V50 and MLD. V5 remained as a significant predictive factor for RIPE and symptomatic RIPE (*p* = 0.007 and 0.022) after adjusting for race, gender and histology. To include, the incidence of RIPE is notable. Whole lung V5 appeared to be the most significant independent risk factor for symptomatic RIPE.

## INTRODUCTION

Thoracic radiation therapy (TRT) is an important component of multi-modality treatment for breast cancer, esophageal cancer, and lung cancer including small cell and non-small cell lung cancer (NSCLC) [1, 2]. TRT can improve survival rates for patients with inoperable stage I or II NSCLC [2–4] and limited stage small cell lung cancer [5]. TRT is a mainstay treatment in combined multi-modalities of surgery and chemotherapy for breast cancer, esophagus cancer, and NSCLC [6–9]. Combination of targeted therapies including immunotherapies with radiation may also significantly improve patient survival in NSCLC [10, 11].

Pulmonary toxicity is a known concern for the use of TRT [12–20]. The total dose of radiation, fraction sizes, volume of lung exposure, and the concurrence of chemotherapeutic treatments have been shown to be factors for toxicity to the lung [13, 21, 22]. Radiation-induced lung injury includes bronchial stenosis, lung edema, pleural effusions, fibrosis, and pneumonitis [23]. As a late effect after TRT, pleural effusion is often induced by inflammation and immune cells such as macrophages and CD8^+^ T cells. Radiation-induced lung toxicity (pneumonitis and fibrosis) occurs in 10% to 30% of patients following TRT [24, 25]. Interstitial pulmonary effects are observed in patients undergoing radiotherapy for bone marrow transplantation [26–29]. Previous partial-volume irradiation parameters were derived from literature reviews and the experience of radiation oncologists [20, 30]. The tolerance doses (TD) for less than 5% risk of lung injury within 5 years of radiation exposure for 1/3, 2/3 or 3/3 of lung volume were empirically considered to be 45, 30, and 17.5 Gy, respectively [20]. Recently, dosimetric factors such as V20 and mean lung dose have been developed to assess the risk of symptomatic radiation-induced lung toxicity (RILT), primarily the development of pneumonitis and fibrosis [22, 31, 32].

To date, a majority of studies investigating risk for lung toxicity in response to radiation have focused primarily on development of pneumonitis and fibrosis [30]. Pleural effusions are frequent manifestations of a variety of systemic and local diseases and are readily and frequently detected on chest radiographs. The appearance of pleural fluid *in vivo* depends on chest wall and lung elasticity and on pressure relationships. Pleural effusion can also develop after radiation damage to the lungs and is considered to be one of the most common late toxicities after TRT [33–35]. Although pleural effusions have been recognized as an adverse effect of radiation exposure for over 50 years, the frequency and risk factors have not been systematically studied during the last two decades [36]. We investigated the rate of thoracic radiation-induced pleural effusion (RIPE) and its risk factors for RIPE in patients with lung cancer.

## MATERIALS AND METHODS

### Patients

#### Patient eligibility

Lung cancer patients treated with TRT between January 2004 and Dec 2014 from two centers (Augusta University and University of Michigan) were reviewed retrospectively on the basis of: pathologically confirmed non-small cell lung cancer, treatment of thoracic RT ± chemotherapy, available computed tomography (CT) scan or PET/CT for treatment planning and dosimetric analysis, 6-month clinical follow-up after RT, with no evidence of local disease progression and initial Eastern Cooperative Oncology Group performance status (ECOG) ≤ 2. Clinical staging was classified according to the American Joint Commission on Cancer staging system, 7th edition [37]. This research was part of an Institutional Review Board approved retrospective study.

### Treatment planning and follow-up

Patients were treated with conventionally fractionated (1.8 or 2.0 Gy/fraction) using 6-, 10-, or 18-MV photons. Most patients were treated with ≥ 60 Gy in 30 fractions over 6 weeks. All patients underwent three-dimensional conformal radiation therapy (3D-CRT) with a CT simulation with a slide thickness of 3.0-5.0 mm. The dose-volume histograms (DVH) parameters analyzed included mean total lung dose (MLD), and volumes of total lung receiving 5 to 55 Gy for each 5 Gy (V5, V10, V15, V20, V25, V30, V35, V40, V45, V50, V55, respectively). Total lung excluding the main bronchus and gross tumor volume was delineated for lung dosimetry computation.

Chemotherapy regimens were mainly based on carboplatin with paclitaxel, or cisplatin with etoposide. Patients were followed per standard of practice. At follow-up, a history, physical examination, and CT of chest or PET-CT were obtained. Chest CT scans and PET/CT were reviewed to evaluate RIPE. Adverse events were graded retrospectively according to the National Cancer Institute-Common Toxicity Criteria Version, version 4.0. Symptomatic effusion was defined as effusion ≥ grade 2. The following clinical factors were investigated in relation to RIPE: age, gender, race, ECOG score, histology, clinical stage, smoking status, chemotherapy, and radiation dose.

### Evaluation of pleural effusion

**Figure 1:**
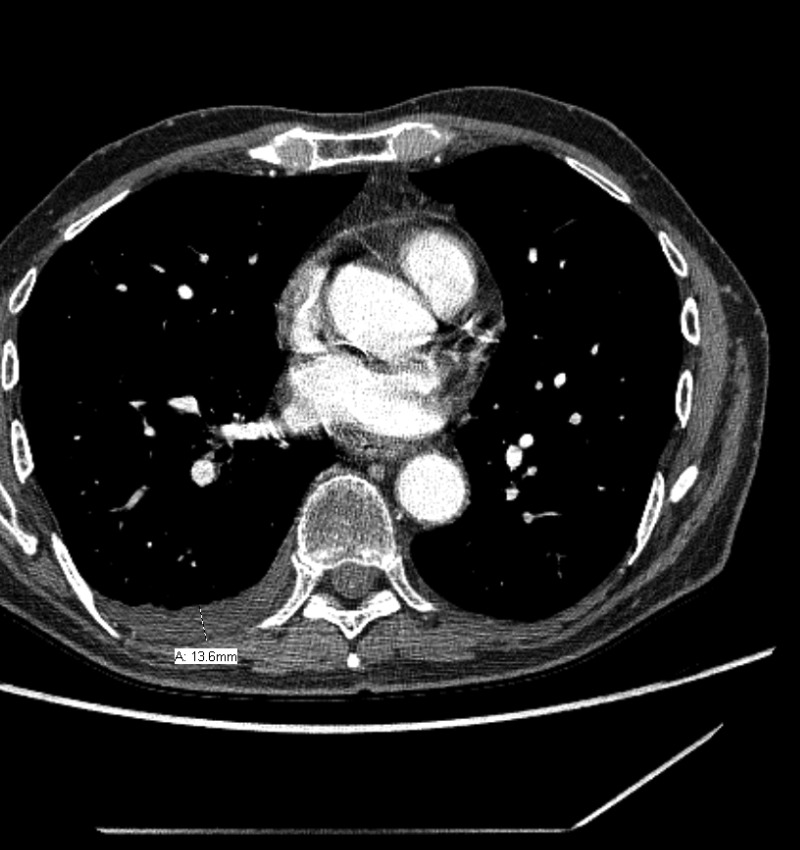
Example measurement for radiation induced pleural effusion

The diagnosis of pleural effusion was independently assessed by one physician, and was spot checked by another physician. Several previous studies reported models based on simple measurement from the chest radiographs to estimate the volume of pleural effusions [38–41]. Patients with fluid loculation were not excluded. We selected the model from Hazlinger, *et al.* [40], who reported the dimension of effusion depth as the best planar measurement which was significantly correlated with the actual PE. The model described by Hazlinger, et al., estimates the PE volumes from CT scans using the formula: the volume of PE = 0.365 × b^3^ – 4.529 × b^2^ + 159.723 × b – 88.377, where b was the depth measured perpendicularly to the parietal pleura on transversal CT scan where the greatest depth was found by scrolling through all the images [40]. An example of measurement is shown in Figure 1. New pleural effusion or increased volume of PE after TRT without evidence of tumor progression was considered to be RIPE. To determine the most relevant dose-volume parameter, a receiver operator characteristic (ROC) curve was constructed. The Youden Index was used as the optimal cutoff from the curve, which is defined as sensitivity + specificity-1.

### Statistical analysis

Time to RIPE was calculated from the last day of TRT to the date at which pleural effusion was observed on the follow-up scan. Patients without pleural effusion (PE) were censored at the last follow-up or death. The overall survival (OS) was defined as the interval between the date of treatment initiation and the date of death, or time of the last follow-up for patients still alive, which was estimated using the Kaplan-Meier method. The logistic regression model was used for multivariate analysis. All analyses were two-sided and differences were considered statistically significant at *p* < 0.05. Statistical analyses were performed employing SPSS, version 19.0 (SPSS Inc, Chicago IL).

## RESULTS

### Patient characteristics

Of 806 patients treated with TRT between 2004 and 2013, 175 with post-treatment imaging available in our electronic medical system and with no evidence of thoracic disease progression were included in this study. The characteristics of the eligible patients were summarized in Table 1. The median OS was 20.5 (range, 6.37–113.2) months. Concurrent chemotherapy was administered to 119 patients.

**Table 1:** Patient characteristics

Characteristics	Subgroup	No. Patients (%)
Age (years)	≤ 65	98 (56.0)
	≥ 65	77 (44.0)
Gender	Male	125 (71.4)
	Female	50 (28.6)
Race	Caucasian	142 (81.1)
	African American	33 (18.9)
Histology	Squamous cell carcinoma	47 (26.9)
	Adenocarcinoma	57 (32.6)
	Others	71 (40.5)
Stage	I	28 (16.0)
	II	32 (18.3)
	III	115 (65.7)
Smoking status	Non-smoker	16 (9.1)
	Former smoker	88 (50.3)
	Current smoker	71 (40.6)
Concurrent chemotherapy	Yes	119 (68.0)
	No	56 (32.0)
Radiation dose (Gy)	≥ 60	123 (70.3)
	< 60	52 (29.7)

Baseline demographic and clinical characteristics of the patients at diagnosis.

### Incidence of radiation induced pleural effusion

RIPE of any grade developed in 51 (29.1%) patients, in which 39 patients had newly developed PE after TRT without disease progression and the remaining 12 patients had PE increases after TRT. The median (range) change of PE depth was 13.6 (2–90.6) mm, and the median (range) change of estimate PE volume was 2164.3 (215.9–248,649) ml. The median (range) RIPE interval from end of TRT was 3.7 (0.6–18.0) months. Forty patients developed symptomatic RIPE; 24 of these patients (60.0%) presented with chest pain; 12 (30.0%) suffered from cough; and 18 (45.0%) had shortness of breath or dyspnea. The actuarial incidence of RIPE at 1 and 2 years for those 51 patients was 88.8% and 11.2%, respectively. The RIPE rates of the two institutions in all patients were 22.6% and 38.6% with a borderline significance in difference (*p* = 0.055).

### Risk factor analysis for patient characteristics

The logistic regression analysis of risk factors for RIPE is shown in Table 2. In all 175 patients, only gender and race were significantly correlated with the occurrence of RIPE: Female (OR = 0.380, 95% CI: 0.156–0.926, *p* = 0.033) and Caucasian race (OR = 3.519, 95% CI: 1.327–9.336, *p* = 0.011) had lower risk of RIPE. Stage and concurrent chemotherapy were correlated with the occurrence of RIPE with a borderline significance (OR = 1.665, *p* = 0.069 for advanced stage and OR = 2.580, *p* = 0.080, for receiving concurrent chemotherapy).

**Table 2:** Multivariate analysis of patient characteristics as risk factors

Characteristics	OR	95%CI	*P*
Age (years)	1.178	0.528–2.629	0.690
Gender	0.380	0.156–0.926	0.033
Race	3.519	1.327–9.336	0.011
Histology	0.705	0.447–1.112	0.132
Stage	1.665	0.961–2.883	0.069
Smoking status	0.905	0.493–1.664	0.749
Concurrent chemotherapy	2.508	0.897–7.014	0.080
Radiation dose	0.830	0.298–2.314	0.721

### Lung volume exposure correlation with development of RIPE

**Figure 2:**
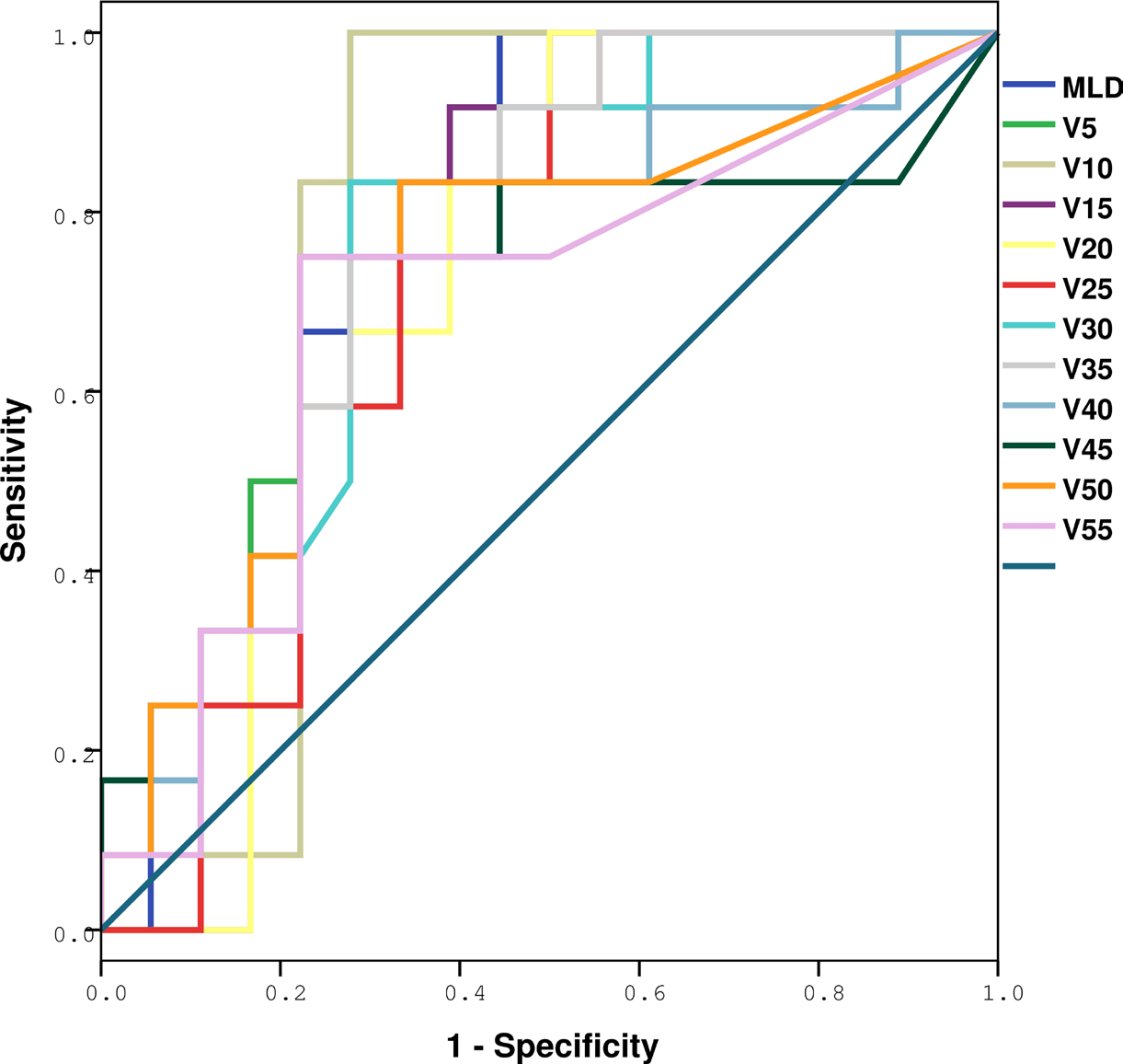
ROC analysis of various dosimetric factors with RIPE Definition of abbreviations: ROC = receiver operating characteristic curves; RIPE = radiation-induced pleural effusion, Vx = Volume of total lung received more than x Gy.

**Figure 3:**
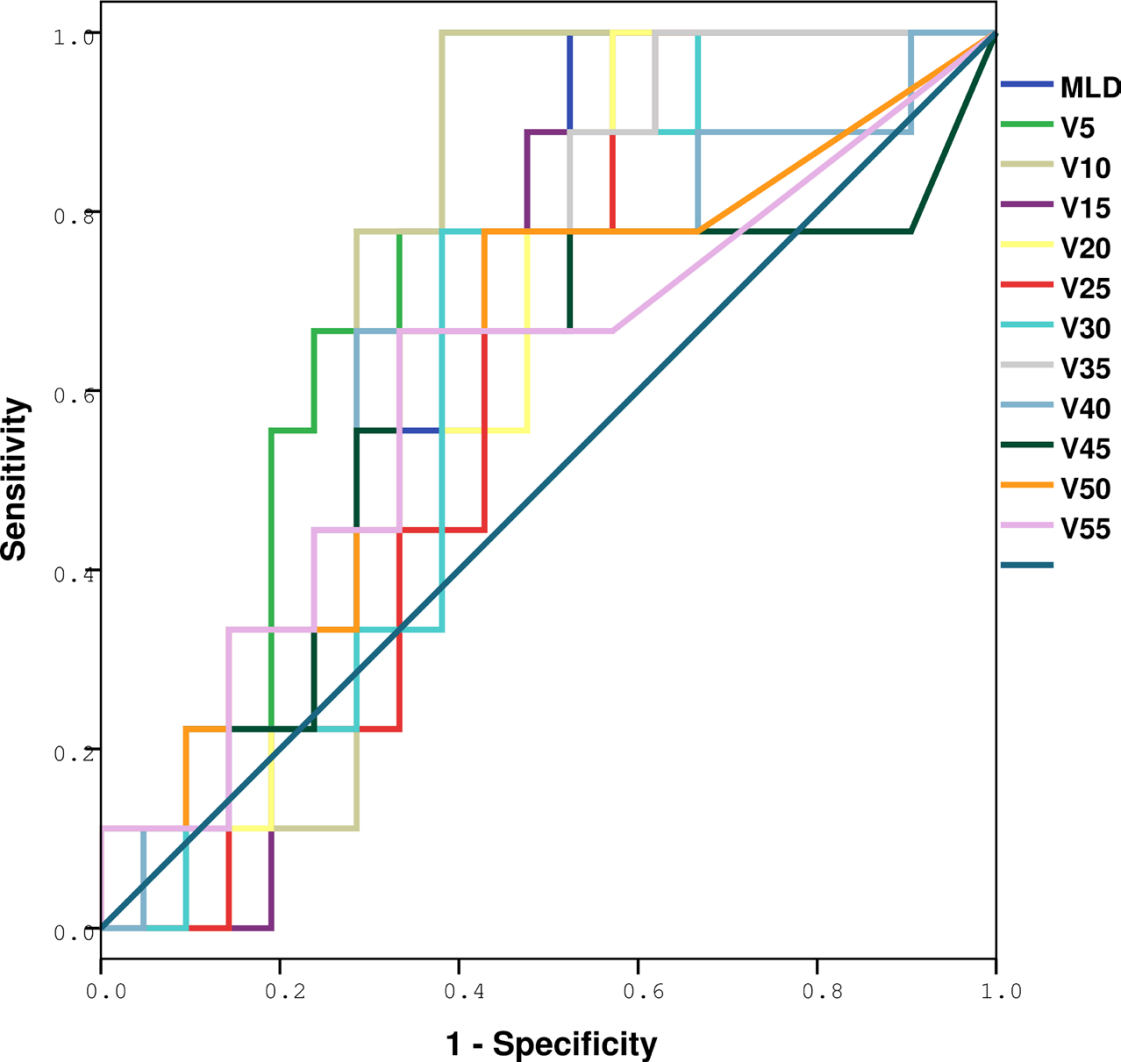
ROC analysis of various dosimetric factors with symptomatic RIPE Definition of abbreviations: ROC = receiver operating characteristic curves; RIPE = radiation-induced pleural effusion.

The ROC curve analysis results are shown in Figures 2 and 3, and Tables 3 and 4. The whole lung V5, V10, V15, V20, V25, V30, V35, V40, V50 and MLD were significantly higher in patients with RIPE than in those without RIPE (*p* = 0.007, 0.011, 0.028, 0.047, 0.038, 0.019, 0.013, 0.034, 0.040 and 0.034), however only V5 remained as a significant predictive factor for symptomatic RIPE (*p* = 0.022) after adjusting for race, age and histology, with the largest area under the ROC curve (AUC = 0.767). For patients with symptomatic RIPE, using a cutoff of 41.5% for V5, the sensitivity and specificity were 100% and 38.1%, respectively.

**Table 3:** Dose-volume histogram and mean lung dose prediction of any radiation-induced pleural effusions by ROC and AUC

Variables	AUC from ROC	SE	95% CI	*p*
V5	0.796	0.086	0.627–0.966	0.007
V10	0.778	0.094	0.593–0.963	0.011
V15	0.741	0.094	0.557–0.924	0.028
V20	0.718	0.095	0.531–0.904	0.047
V25	0.727	0.093	0.544–0.910	0.038
V30	0.757	0.089	0.583–0.931	0.019
V35	0.773	0.085	0.607–0.940	0.013
V40	0.731	0.097	0.542–0.921	0.034
V45	0.704	0.106	0.495–0.912	0.063
V50	0.731	0.098	0.539–0.924	0.034
V55	0.692	0.104	0.489–0.895	0.079
MLD	0.773	0.086	0.604–0.942	0.013

Definition of abbreviations: ROC = receiver operating characteristic curves; AUC = area under the curve; SE = standard error of the mean; CI = confidence interval.

**Table 4:** Dose-volume histogram and mean lung dose prediction of symptomatic radiation-induced pleural effusions by ROC and AUC

Variables	AUC from ROC	SE	95% CI	*p*
V5	0.767	0.085	0.601–0.934	0.022
V10	0.714	0.094	0.530–0.899	0.067
V15	0.635	0.099	0.441–0.828	0.248
V20	0.614	0.100	0.417–0.810	0.331
V25	0.619	0.101	0.422–0.817	0.309
V30	0.640	0.100	0.444–0.837	0.230
V35	0.677	0.097	0.487–0.867	0.129
V40	0.624	0.111	0.407–0.842	0.288
V45	0.593	0.123	0.351–0.834	0.428
V50	0.635	0.114	0.411–0.858	0.248
V55	0.606	0.118	0.375–0.837	0.365
MLD	0.688	0.095	0.510–0.874	0.108

Definition of abbreviations: ROC = receiver operating characteristic curves; AUC = area under the curve; SE = standard error of the mean; CI = confidence interval.

### Correlation of RIPE with overall survival

**Figure 4:**
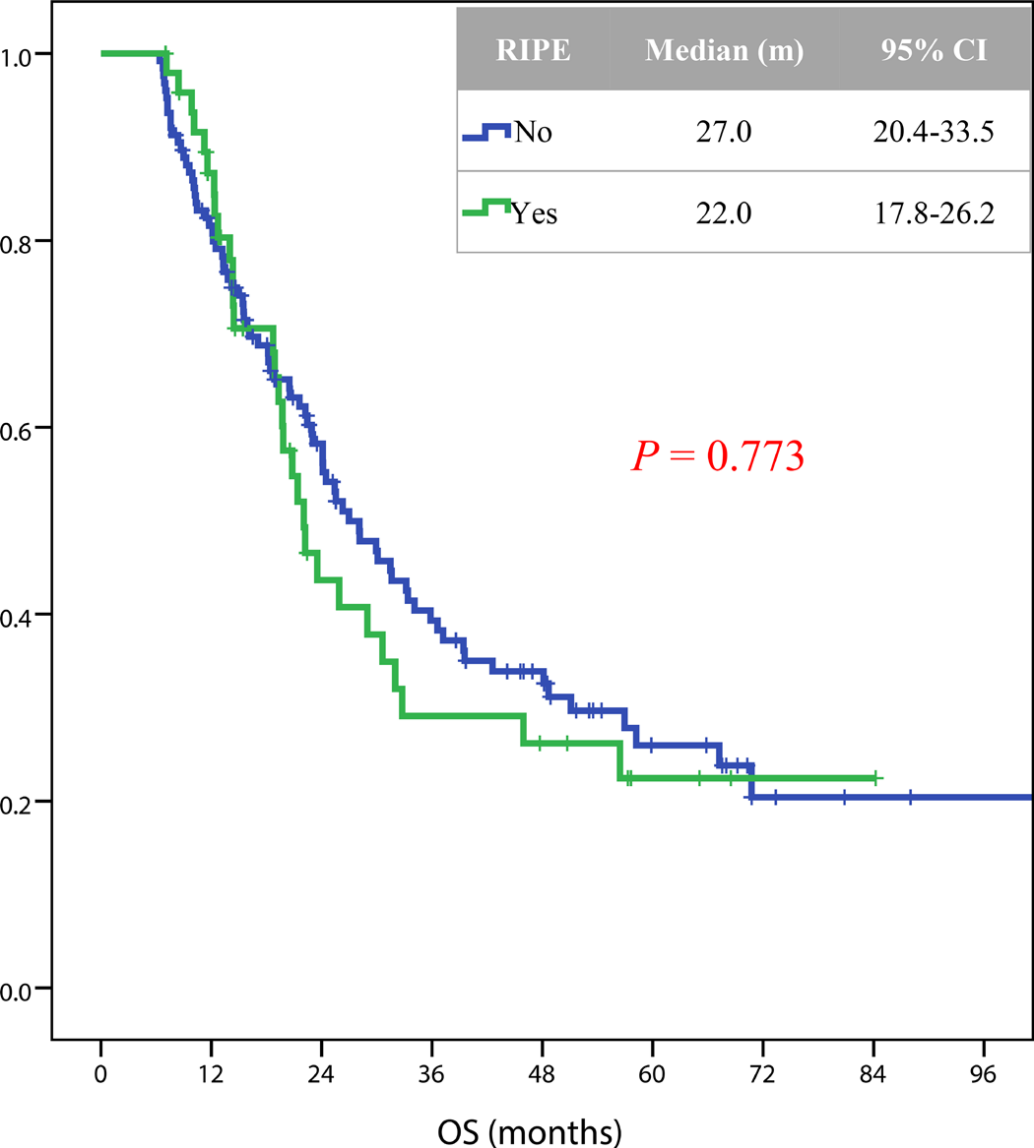
Kaplan-Meier estimates of OS in 205 patients with or without RIPE after radiotherapy Definition of abbreviations: OS = overall survival; RIPE = radiation-induced pleural effusion.

In total, 109 patients died during a median follow up of 19.5 months. RIPE was not significantly correlated with the overall survival (Figure 3). The median (95% CI) overall survival rates for patients with or without RIPE were 22.0 (17.8–26.2) and 27.0 (20.4–33.5) months, respectively (*p* = 0.773) (Figure 4).

## DISCUSSION

This study demonstrated that RIPE occurred in 29.1% of patients treated with TRT. In 80% of patients with RIPE, there was an association with significant symptoms, such as chest pain and shortness of breath. Lung dosimetric factors were significantly greater for patients with RIPE, but only V5 was statistically significant for symptomatic RIPE in this series. Interestingly, Caucasian race and squamous cell tumor histology were each associated with a lower risk for RIPE.

Dose-volume parameters that predict the occurrence of RIPE include the dose of radiation and the volume of the lung exposed to radiation. Our study indicated that although patients with RIPE had exposures from V5-V55, only V5 was a significant risk factor for RIPE. According to the latest National Comprehensive Cancer Network guidelines, the limit of lung volume receiving more than 20 Gy and 5 Gy (V20 and V5) were 35% and 65%, respectively [35]. The data used to generate these guidelines were largely obtained from studies of symptomatic radiation pneumonitis, and did not take into consideration symptomatic RIPE as a late side effect. In our current study, we found that < 41.5% could be a threshold to avoid a high risk of RIPE, suggesting that potential benefit of better treatment plans with more stringent limit of V5 to decrease the risk of late symptomatic RIPE. Future studies should validate our findings by defining pleural surface and generate the pleural dosimetry as one of organs at risk for RT planning in the clinic and consider RIPE in animal studies.

Our current study found that a significant number of patients with pleural effusions also had chest pain, dry cough, and dyspnea, in agreement with previous findings [42]. The management of pleural effusions depends upon the underlying etiology [42]. When associated with disease states, pleural effusions, both benign effusions and cancer-associated, are correlated with high mortality [43, 44]. Pleural effusions are a significant risk factor for the treatment of thoracic cancers [45], and the presence of pleural effusions with lung cancer can preclude curative surgery [45].

This etiology of pleural effusions following radiation treatment of thoracic cancers remained to be studied. The presence of pleural effusions in malignancy often correlate with changes in the disease course and predict changes in responses to cancer therapies [44, 45]. However, both non-inflammatory and inflammatory reactions to radiation can cause increased fluid in the pleural cavity [42, 46]. Normally, the pleural surface is surrounded by ∼15 ml of acellular, clear fluid that acts as a lubricant for the thoracic cavity. Post-RT pleural effusion could also be from benign pleural causes such as cardiac, kidney or liver failure, infections (such as tuberculosis, pneumonia, lung infarcts, lung abscess, and bronchiectasis), systemic disorders (such as rheumatoid arthritis, systemic lupus erythematosus, uremia, or systemic infections), trauma, or radiation therapy.

The biology or cytopathology of pleural effusions following ionizing radiotherapy has also not been systematically studied [47]. One study examined specimens from 55 irradiated patients with pleural effusions and identified bizarre cells, but no other distinctive cytologic changes [47]. Increased secretion into the pleural space is thought to involve a wide variety of cells, including mesothelial, endothelial, myeloid, and lymph cells [44]; in the case of malignant pleural effusions (MPEs), tumor cells are also thought to contribute significantly to secretions [44]. Tumor volumes and tumor metabolic activities, as well as the unique tumor secretome have been demonstrated to affect MPEs, which are a common complication of advanced malignancy [45]. A variety of biochemical markers have been identified in MPEs, including pH, lactate dehydrogenase, neutrophil-to-lymphocyte ratio, and the protein surviving, and attempts have been made to correlate these factors with patient prognosis [45]. Reasons of MPE cannot explain RIPE as we only elected those without tumor progression for radiation induced toxicity.

Murine model studies of the effects of thoracic irradiation have demonstrated that pleural effusions occur in a number of rodent models including rats and some strains of mice [35, 48]. Whole thorax irradiation was also demonstrated to induce hypoxic respiratory failure, cardiac injury, and pleural effusions in Wistar rats [35]. However, recent recommendations have been to select murine strains that do not exhibit pleural effusions in studies of radiation-induced lung injury [35, 48]. In light of our current finding, the study of murine models that do develop RIPE is essential to the understanding of the complexity of radiation-induced injuries to the thoracic tissues in patients. The use of murine models with RIPE would additionally aid in the understanding of the mechanism(s) of pleural effusion in response to ionizing radiation as well as the identification of cytologic alterations that occur. Considering the relative high rate of RIPE and its clinical significance, future studies are needed to understand the mechanism in both animal models and patients.

## Abbreviations

TRT: Thoracic radiation therapy
NSCLC: non-small cell lung cancer
RILT: radiation-induced lung toxicity
RIPE: radiation-induced pleural effusion
ECOG: Eastern Cooperative Oncology Group performance status
3D-CRT: three-dimensional conformal radiation therapy
DVH: dose-volume histograms
MLD: mean total lung dose
ROC: receiver operator characteristic
